# Supplementary material for: Ergodic Transition in a Simple Model of the Continuous Double Auction
Source: PLoS One. 2014 Feb 18;9(2):e88095. doi: 10.1371/journal.pone.0088095 (PMC3928121; doi:10.1371/journal.pone.0088095)
Supplement: File S1 — Supporting information. (PDF) [file pone.0088095.s001.pdf]

Ergodic transition in a simple model of the continuous double auction

Supplementary code

The MATLAB® code, consisting of the functions `cda.m` and `order.m` and used for simulations of the double-auction model is listed below.

```
function cda(N,n,ratio,nevents,nruns)
% INPUT arguments
%   N <- upper limit for prices
%   n <- size of interval for placing orders around bestask/bestbid/last price
%   ratio <- lambda/mu; ratio between limit orders and market orders arrival rate;
%            can be scalar or vector
%   nevents <- number of events of placing either limit or market order
%   nruns <- number of Monte Carlo steps
%
% OUTPUT arguments (saved in .mat file):
%   descriptive statistics - mean, standard deviation, skewness,
%       kurtosis of series of returns and autocorrelation at the
%       first lag of absolute returns (c_1)
%   ret - the last series of returns
%   P - the last series of prices

%   initial price : ceil(N/2)
%   Options for calculating the returns - go to line 122
%       default: logarithmic return

tic;

ma = 3; % market sell order rate
mb = 3; % market buy

rs = size(ratio); rs = rs(2);

retmean = zeros(nruns,rs);
retstd = zeros(nruns,rs);
retskew = zeros(nruns,rs);
retkurt = zeros(nruns,rs);
acorr = zeros(nruns,rs);

for r = 1:rs % loop for different ratios
    lb = ratio(r)*ma;
    la = ratio(r)*mb;

    for i = 1:nruns

        % state of the order book
        X = zeros(1,N);

        % initialization
        price = ceil(N/2);
```

```
P = [0 price];

bestbid = 0;
bestask = 0;

k = 0; T = 0;

while k < nevents
    k = k + 1;

    [event, t] = order(la,lb,ma,mb); % choose type of the order
    T = T + t;

    if event == 1 % limit order bid

        if bestask==0
            if price <= n
                p = randi(price);
            else
                p = price - (randi(n+1) - 1);
            end
        else
            if bestask == 1
                continue
            elseif bestask <= n
                p = bestask - randi(bestask-1);
            else
                p = bestask - randi(n);
            end
        end
        X(p) = X(p) - 1; % new bid

    elseif event == 2 % limit order ask

        if bestbid==0
            if price > N - n
               p = price + randi(N-price+1) - 1;
            else
               p = price + randi(n+1) - 1;
             end
        else
            if bestbid == N
                continue
            elseif bestbid > N - n
                p = bestbid + randi(N-bestbid);
            else
                p = bestbid + randi(n);
            end
        end
        X(p) = X(p) + 1; % new ask

    elseif event == 3 % market buy
        if bestask~=0
            X(bestask) = X(bestask) - 1;
            price = bestask;
            P = [P; T price];
        end
    else % market sell
        if bestbid~=0
            X(bestbid) = X(bestbid) + 1 ;
            price = bestbid;
            P = [P; T price];
```

```
                end
            end

            % update bestask, bestbid
            bestask = find(X>0,1);
            if isempty(bestask)==1
                bestask = 0;
            end
            bestbid = find(X<0,1,'last');
            if isempty(bestbid)==1
                bestbid = 0;
            end
        end

        ret = [diff(log(P(:,2)))]; % log-returns
%         ret = [diff(P(:,2))./P(1:end-1,2)]; % returns
%         ret = [diff((P(:,2)))]; % price differences

        retmean(i,r) = mean(ret);
        retstd(i,r) = std(ret);
        retskew(i,r) = skewness(ret);
        retkurt(i,r) = kurtosis(ret);
        acl1 = autocorr(abs(ret),1);
        acorr(i,r) = acl1(2);

   end
end

save(['returns_N',num2str(N),'n',num2str(n),'r',num2str(ratio),'.mat'],...
    'retmean','retstd','retskew','retkurt','acorr','ratio','P','ret');

toc;

end
```

The additional function `order` called by the main function is the following.

```
function [event, t] = order(la,lb,ma,mb)

c = la + lb + ma + mb;
x = rand(1);

if x < lb/c
    event = 1; % limit bid
    t = -log(rand(1))/c;
elseif x < (lb + la)/c
    event = 2; % limit ask
    t = -log(rand(1))/c;
elseif x < (lb+la+mb)/c
    event = 3; % market buy
    t = -log(rand(1))/c;
else
    event = 4; % market sell
    t = -log(rand(1))/c;
end
end
```
